# Profiles of Accelerometry-Derived Physical Activity Are Related to Perceived Physical Fatigability in Older Adults

**DOI:** 10.3390/s21051718

**Published:** 2021-03-02

**Authors:** Jessica L. Graves, Yujia (Susanna) Qiao, Kyle D. Moored, Robert M. Boudreau, Elizabeth M. Venditti, Robert T. Krafty, Eric J. Shiroma, Jaroslaw Harezlak, Nancy W. Glynn

**Affiliations:** 1 Department of Epidemiology, University of Pittsburgh, Pittsburgh, PA 15261, USA; jeg143@pitt.edu (J.L.G.); susannaqiao@pitt.edu (Y.Q.); kdm77@pitt.edu (K.D.M.); boudreaur@edc.pitt.edu (R.M.B); 2 Department of Psychiatry, University of Pittsburgh, Pittsburgh, PA 15213, USA; vendittiem@upmc.edu; 3 Department of Biostatistics and Bioinformatics, Emory University, Atlanta, GA 30322, USA; robert.t.krafty@emory.edu; 4 Laboratory of Epidemiology and Population Sciences, National Institute on Aging, Baltimore, MD 21225, USA; eric.shiroma@nih.gov; 5 Department of Epidemiology and Biostatistics, School of Public Health-Bloomington, Indiana University, Bloomington, IN 47405, USA; harezlak@iu.edu

**Keywords:** fatigue, rest–activity rhythm, older adults

## Abstract

Physical activity (PA) is associated with greater fatigability in older adults; little is known about magnitude, shape, timing and variability of the entire 24-h rest–activity rhythm (RAR) associated with fatigability. We identified which features of the 24-h RAR pattern were independently and jointly associated with greater perceived physical fatigability (Pittsburgh Fatigability Scale, PFS, 0–50) in older adults (*n* = 181, 71.3 ± 6.7 years). RARs were characterized using anti-logistic extended cosine models and 4-h intervals of PA means and standard deviations across days. A K-means clustering algorithm approach identified four profiles of RAR features: “Less Active/Robust”, “Earlier Risers”, “More Active/Robust” and “Later RAR”. Quantile regression tested associations of each RAR feature/profile on median PFS adjusted for age, sex, race, body mass index and depression symptomatology. Later rise times (up mesor; β = 1.38, *p* = 0.01) and timing of midpoint of activity (acrophase; β = 1.29, *p* = 0.01) were associated with higher PFS scores. Lower PA between 4 a.m. and 8 a.m. was associated with higher PFS scores (β = −4.50, *p* = 0.03). “Less Active/Robust” (β = 6.14, *p* = 0.01) and “Later RAR” (β = 3.53, *p* = 0.01) patterns were associated with higher PFS scores compared to “Earlier Risers”. Greater physical fatigability in older adults was associated with dampened, more variable, and later RARs. This work can guide development of interventions aimed at modifying RARs to reduce fatigability in older adults.

## 1. Introduction

Perceived physical fatigability, defined as an individual’s susceptibility to fatigue anchored to activities of specified intensity and duration [1,2], is prevalent with advanced age, such that about 25% of older adults aged 60–69 and approximately 82% of those at least 90 years of age report greater fatigability [3]. Physical fatigability has become an increasingly relevant age-sensitive construct and numerous studies have shown it is associated with a variety of health outcomes, including slower gait speed and functional decline, suggesting that fatigue plays a role along the disablement pathway [4,5]. In addition to physical function, greater physical fatigability has been associated with other indicators of health status, such as cognition, chronic low-grade inflammation, subclinical peripheral artery disease and greater cardiovascular disease burden [2]. Collectively, these findings demonstrate that physical fatigability is a key marker of phenotypic aging.

Perceived physical fatigability may influence markers of phenotypic aging through a complex, bidirectional relationship with free-living activity, both contributing to decline in physical function. Greater fatigability has been associated with decreased, and more fragmented, activity patterns in older adults [6,7,8]. Wanigatunga et al. [8] found that greater performance-based perceived physical fatigability was associated with lower physical activity, particularly between the hours of 8:00 a.m. to 8:00 p.m. in the Baltimore Longitudinal Study of Aging (BLSA) cohort. Additionally, Schrack et al. [6] found that fragmentation of physical activity, or the active-to-sedentary transition probability (ASTP), was associated with greater fatigability in BLSA. This suggests that not only the amount, but also the patterns of physical activity are related to fatigability in later life. Analytic techniques that capture both the amount and patterns of activity (e.g., RARs) simultaneously may be especially useful in clarifying which activity characteristics influence fatigability and may enable us to identify profiles of RARs in older adults at risk of future functional limitations.

These prior studies also capture the important role that accelerometry-derived measures of physical activity can play in understanding physical fatigability. However, in these studies, physical activity was defined as the outcome, which may not wholly reflect the mechanistic relationship between physical activity and perceived fatigability. Recent work examining the bidirectional associations of physical activity and perceived fatigability using the Long Life Family Study found that there was mediation of physical activity on gait speed through fatigability, but not vice versa [9]. Thus, it is important to re-examine these relationships treating physical activity patterns as the independent variable, as doing so may more accurately reflect the aging-related disablement pathway.

Furthermore, while Schrack et al. [6] highlighted the importance of understanding the specific role of variability and fragmentation of activity on fatigability, their approach had limited ability to identify when in the activity cycle fragmentation occurs, thus exploring alternative approaches using accelerometry is warranted. Circadian rhythms are approximately 24-h endogenous cycles that help to regulate both biology and behavior [10]. Perhaps the most salient of these circadian rhythms is the pattern of rest and activity, called the rest–activity rhythm (RAR). The RAR is the downstream behavioral manifestation of the alignment or misalignment of several circadian processes. Older adults are susceptible to age-related changes in RARs [11,12]. These changes include: diminished melatonin and cortisol levels, hormones critical for dictating the strength of the RAR [13] and shifts in the timing of circadian patterns [14]. Studies of free-living accelerometry-derived RARs have shown that features of RARs in older men are associated with a variety of negative health outcomes. Namely, irregular RARs were associated with increased risk for all-cause and cardiovascular disease-related mortality [15], falls [16], and cognitive decline [17]. Similarly, epidemiologic studies in older women showed that weakened RAR patterns were associated with increased mortality risk [18].

Although previous studies emphasized the impact of daytime activity on physical fatigability, to our knowledge, no studies have leveraged circadian science methods, such as flexible nonlinear modeling, to explore how the magnitude, shape, timing, and variability of the entire 24-h free-living RARs are associated with perceived physical fatigability in older adults. Older adults > 65 years in the United States are the least physical active adults of any age group [19] and as many as 70% of older adults report chronic sleep disturbances [14], making them particularly vulnerable to the negative effects of reduced physical activity and fragmented RARs. Additionally, physical activity is an established and modifiable correlate of fatigability [9,20]. RAR analysis could provide specific insights as to which specific features of overall patterns of rest and activity, or combinations of features, are most strongly associated with greater perceived physical fatigability. In turn, this work could enable future researchers and clinicians to more effectively identify those at risk of greater fatigability and inform interventions to prevention or delay of functional decline. Since RARs and physical activity may jointly influence each other, it is important to consider RARs as a key target for future intervention studies and may be a useful biomarker in identifying at-risk populations.

Therefore, to expand upon this growing area of research, the aim of this study was to identify how both individual and combinations of features of accelerometry-derived free-living RARs (e.g., magnitude of activity, shape of the RAR, activity timing, and robustness of the RAR) were associated with perceived physical fatigability in a sample of older adults. 

## 2. Materials and Methods

### 2.1. Study Sample

This study leveraged accelerometry and fatigability data collected in two existing cohorts of community-based older adults. Briefly, the Mobility and Vitality Lifestyle Program (MOVEUP) was a non-randomized, 13-month behavioral weight management trial in older adults aged 60–75 years (68.2 + 4.1 years, 86% female) who were obese or overweight [21]. Inclusion criteria were based on age, body mass index (BMI) of 27–45 kg/m^2^, ambulatory (with the use of a cane permitted), and cognitively intact. Exclusion criteria includes: active treatment for cancer, overnight hospitalization in the past 6 months, uncontrolled diabetes mellitus or hypertension, and any limitations that might preclude participation in the program (outside of basic accommodations), such as significant cognitive impairment or visual or hearing loss. The primary outcome for the MOVEUP study was a change in physical function at 13 months (post-intervention). Secondary outcomes included weight change, accelerometry-based, and self-reported physical activity among other items. For these analyses, baseline data were used. The Developmental Epidemiologic Cohort Study (DECOS) was a cross-sectional study examining the impact of accelerometry wear location on the quantification of physical activity and sedentary behaviors among older adults 70–92 years (78.6 + 5.7 years, 60% female) [22]. Exclusion criteria for DECOS included any self-reported health contraindication to physical testing and the inability to perform basic mobility tasks (e.g., severe pain, aching, or stiffness while walking).

### 2.2. Assessment of Exposure, Outcome, Covariates

Assessment of exposure: Free-living RARs were measured using accelerometry. Accelerometers were only allocated to a subsample of participants in the MOVEUP sample (the first 11 sites, *n* = 127), due to budgetary limitations. Of the DECOS sample (*n* = 69), 61 consented to accelerometry data collection. To quantify the entire 24-h RAR, both cohorts were instructed to wear an ActiGraph GT3X+ accelerometer at all times, including during sleep, on the non-dominant wrist for 7 consecutive days. Participants were told to remove their accelerometers during shower, bathing, or swimming. The sampling rate for the ActiGraph GT3X+ was set to 80 Hz (80 observations per second). 

Assessment of the outcome: Perceived physical fatigability was measured using the Pittsburgh Fatigability Scale (PFS) [23]. The 10-item PFS was validated in adults aged ≥ 60 years from two research registries at the University of Pittsburgh. The PFS showed strong internal consistency (Cronbach’s alpha = 0.88) and excellent test–retest reliability (intraclass correlation = 0.86). Concurrent and convergent validity was high against measures of performance fatigability, mobility, physical function, and fitness. The PFS wass a self-administered and participants were asked to rate the fatigue they expect or imagine that they would feel from 0 (“no fatigue”) to 5 (”extreme fatigue”) immediately after performing tasks of specified intensity and duration (e.g., a “leisurely walk for 30 min”). We summed up the items to derive PFS Physical scores (ranging from 0–50), with higher scores denoting greater perceived physical fatigability. A PFS score ≥ 15 has been established as a cut point indicating greater perceived physical fatigability [2,24]. Incomplete PFS scores were imputed based on the method described in Cooper et al. [25]. Five PFS scores were imputed (*n* = 1 MOVEUP; *n* = 4 DECOS).

Covariates: We chose the selected covariates based on prior work by our group regarding their association with perceived physical fatigability [3]. We ascertained age, sex, and race by a self-reported questionnaire. We measured height to the nearest 0.25 cm using a portable stadiometer and weight using a calibrated digital scale; both were used to calculate body mass index (BMI, (weight (kg)/height (m^2^)). The Short Physical Performance Battery (SPPB) was used to evaluate a lower extremity physical function, and included tests of gait speed, standing balance, and chair-stands; SPPB total score (range 0–12) was used in analyses [26]. Self-reported physical activity was measured using the Community Healthy Activities Model Program for Seniors (CHAMPS] questionnaire (MET-min/wk] [27]. Depressive symptomatology was evaluated using the Center of Epidemiologic Studies Depression Scale (CES-D] [28]. 

Accelerometry data cleaning: We extracted raw accelerometry data from each device and converted 80 Hz data into 60-s epoch counts using the ActiLife software. In order to capture complete days of accelerometry data, we truncated accelerometry data to start at the first midnight and to end at the 6th midnight. Participants were then screened for non-wear time using Choi’s algorithm [29]. Through this algorithm, we defined non-wear time as 90 consecutive minutes of zero counts, with an allowance of 2 min of nonzero counts provided there were 30 min of consecutive zeros up and down stream. A valid wear day was any day that consisted of at least 10-h of wear time. Each participant had to have a minimum of 3 valid days in order to be included in the analyzed sample. Under accelerometry data cleaning criteria, the DECOS sample had 57 participants with usable activity data, 54 of which had completed the PFS; the MOVEUP sample had 127 participants with usable activity data and all completed the PFS, making a total analytic sample of 181. 

### 2.3. Statistical Methods

**Figure 1:**
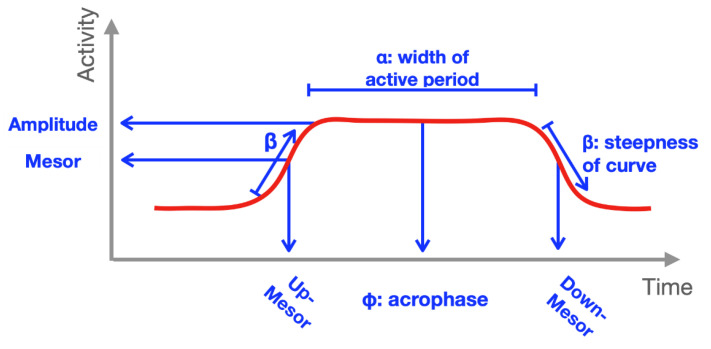
Schematic of parameters estimated from antilogistic extended cosine model and how they impact the size and shape of the rest–activity rhythm.

Estimating activity and RARs: We estimated mean-level RARs using the antilogistic extended cosine model [30]. These models extend the number of parameters in the cosine model to more flexibly capture the characteristics of periodic rest and activity. The antilogistic extended cosine model can be represented as: $f\left(t;\theta \right)=m+\mathrm{amp}*\mathrm{expit}\left(\left\{\beta \left[\cos\left(\left[\frac{t}{r}-\varphi \right]\frac{2\pi }{24}\right)-a\right]\right\}\right)$, where t is time, r is the number of non-overlapping observation epochs within an hour, *expit(x)* = $e^{x}/\left(1+e^{x}\right)$. The antilogistic extended cosine model is indexed by parameters θ=(m, amp, α, β, φ)′. The parameters estimated from this model provide interpretable estimates of the magnitude, shape, timing, and global variability of the RAR. All activity counts were log transformed (counts + 1) prior to modeling. Parameters obtained included: alpha (width), amplitude, acrophase, beta (steepness), mesor (exp(m + amp/2)), up mesor (also known as “estimated rise time”), and the “pseudo-F” statistic (rhythmicity/more variability) (Figure 1). 

Localized measures of RAR attempt to complement parameter estimates from the extended cosine model by calculating means and standard deviations of activity within prespecified time-intervals (e.g., 00:00–04:00, 04:00–08:00, …, 20:00–24:00). Localized measures estimate the time-specific absolute mean activity, the mean of activity across days within a specific time interval, and the standard deviation of activity, which estimates time-specific variability of daily mean-level activity within specific time-intervals across days. Here, localized measures of timing and variability, mean and standard deviations of activity were calculated within 4-h time bin intervals (00:00–04:00, 04:00–08:00, …, 20:00–24:00), for comparability to previous work [8]. Parametric and localized measures were estimated using R package RAR (https://github.com/JessLGraves/RAR accessed on 15 November 2020). 

Statistical analyses: Descriptive analyses identified any cohort characteristics that may have differed based on our outcome of interest (greater perceived physical fatigability) in order to identify any potential confounding variables and characterize the sample. To test group differences, Pearson’s Chi-squared tests for categorical variables and two-sample *t*-tests for continuous variables were used when assumptions of normality held (Kruskal–Wallis test used if normality assumptions are not met).

As continuous PFS Physical scores were positively skewed, quantile regression models adjusting for both a priori defined and suspected confounders estimated the association between PFS Physical scores and individual RAR parameters and means and standard deviations of physical activity within time bins. Quantile regression is well-suited for non-normally distributed continuous variables and was used to estimate quantiles (e.g., 25th, 50th (median), and 75th percentiles) instead of the mean as is done in ordinary least squares regression. We used the median to estimate the central tendency of PFS Physical scores. 

K-means clustering algorithm, an unsupervised clustering approach, was applied to the sample’s standardized extended-cosine RAR parameters using the R package stats. Cluster number validity was confirmed using a variety of diagnostic techniques: Ward’s method, principal components analysis, evaluation of the within sum of squares at multiple cluster assignments, and visualization of cluster assignments along the first two dimensions of the data. We subsequently tested the cluster assignments to see if they were univariately associated with PFS Physical scores using Kruskal–Wallis tests and multivariate quantile regression with adjustment for potential confounders (age, sex, race (white versus non-white), body mass index, and depression symptomatology. Statistical significance was defined as *p*-value < 0.05.

## 3. Results

The participants in the combined sample had a mean age of 71.3 years and were predominately female (79.0%), white (73.9%), educated (79.3% had at least a high school education level), and obese (mean BMI = 32.3 kg/m^2^). A total of 79.3% of those with greater fatigability (PFS scores ≥ 15) were from the MOVEUP sample (*p* < 0.001). Those with greater perceived physical fatigability had worse SPPB scores (*p* = 0.024), higher BMI (*p* < 0.001), slower usual gait speeds (*p* < 0.001), less self-reported physical activity (*p* = 0.025), and worse depressive symptomology (*p* < 0.001) (Table 1, see Supplemental Material Table S1 for Cohen’s D effect sizes). 

Greater perceived physical fatigability was associated with a later acrophase (i.e., time at which activity is at its midpoint, *p* = 0.022) and up mesor (i.e., estimated rise time, *p* = 0.005) (Table 2) and with more than a 0.3 fewer days RAR observation on average (data not shown). Table 2 also shows the results of the separate multivariable quantile (median) regression models where each RAR parameter was included as an individual predictor of median PFS Physical scores, adjusted for age, sex, race, BMI and depressive symptomatology score. Beta (increased steepness of the RAR curve), acrophase and up mesor (later timing) were all significantly and positively associated with median PFS score.

**Figure 2:**
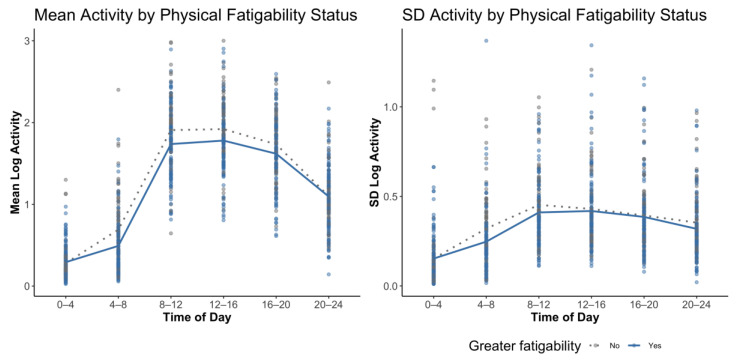
Mean and standard deviation of daily log activity counts stratified by the Pittsburgh Fatigability Scale (PFS) at 4-h time bin intervals. Solid line represents greater perceived physical fatigability (PFS ≥ 15); dotted line represents lesser perceived physical fatigability (PFS < 15).

Figure 2 shows the distribution of means and standard deviations of physical activity across 4-h time bins, stratified by perceived physical fatigability status (PFS score ≥ 15 versus < 15). Qualitatively, we see that those with greater perceived physical fatigability (solid blue line) had an overall dampened activity pattern compared to those with lower fatigability. They also show lower variability (standard deviation) of activity across all time bins. Table 3 shows that mean and standard deviation of activity were associated with PFS Physical scores across 4-h time intervals using multivariate quantile regression. These results show that lower levels of mean level activity between 4:00 a.m. and 8:00 a.m. were associated with higher PFS Physical scores (β = −4.51, *p* = 0.025), adjusted for age, sex, race, BMI and depressive symptomatology score. 

Diagnostics of the k-means clustering algorithm on k = 4 are presented in Figure S1. The dendrogram derived from using the Ward algorithm (based on Euclidean distances between scaled RAR parameters) provides strong evidence that we should select at least three clusters. We can also see that a fourth branch allows for the emergence of an additional distinct class of RARs. Additionally, the within sum of squares (“the Elbow plot”) estimated from alternative values of k did not show a distinct “elbow”, providing evidence that there was not a clear recommended number of clusters based on this selection technique. Additional diagnostic techniques, such as the “scree” plot generated from principal components analysis (PCA) showed that four clusters might be beneficial and might not result in over fitting (due to lack of elbow), and its inclusion results in 91% of variance explained. We visualized how cluster assignments aligned with the first two principal components generated from PCA and saw that four clusters allowed us to capture differences across these dimensions, while also retaining some nuance between them. For example, the “green” cluster captured separations between the “red” and “purple” cluster, allowing for characterization of another cluster of RAR patterns.

**Figure 3:**
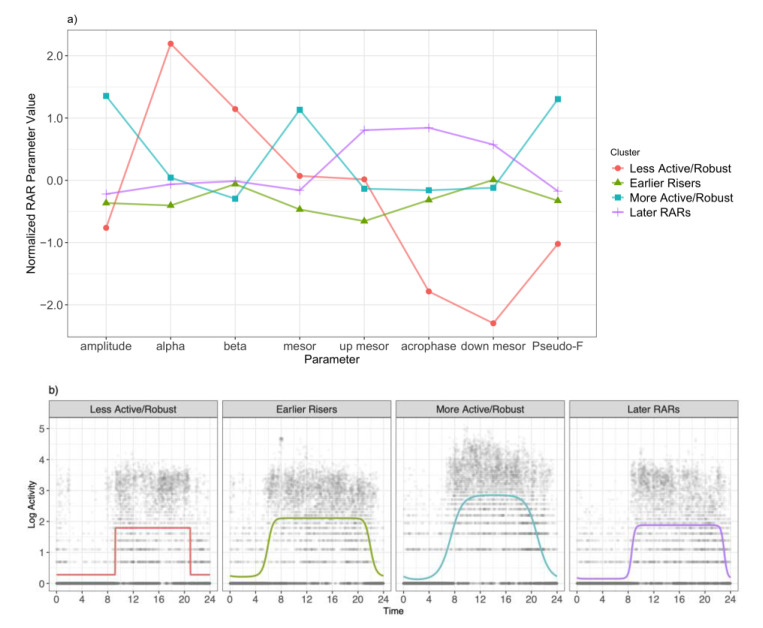
Rest–activity rhythm (RAR) profiles defined by the k-means cluster analysis. (**a**) Each k-means derived cluster’s mean RAR parameter estimates from the antilogistic extended cosine model. (**b**) Example estimated RAR patterns highlighting key differences between each RAR cluster type. Solid lines indicate the predicted RAR based on the extended cosine model; dots represent individual log activity count observations over the study period.

Figure 3a shows the mean normalized RAR parameter estimates for each cluster assignment on a normalized scale. We see that Cluster 1 (“Less Active/Robust”) had higher alpha (or a narrower active period) and a higher beta (steeper transition from rest to active), but lower (earlier) timing, particularly towards the end of their RAR, and less rhythmicity/more variability (lower pseudo-F statistic). Cluster 2 (“Earlier Riser”) is a fairly “average” RAR, with slightly earlier up mesor time. Cluster 3 (“More Active/Robust”) represents RAR patterns with higher magnitudes of activity (higher amplitude and higher mesor) and stronger rhythmicity/less variability (higher pseudo-F statistic). Cluster 4 (“Later RAR”) represents RAR patterns where timing was later in the day as up mesor, acrophase, and down mesor were all larger. Figure 3b shows example RAR patterns that typify the identified RAR profiles.

**Figure 4:**
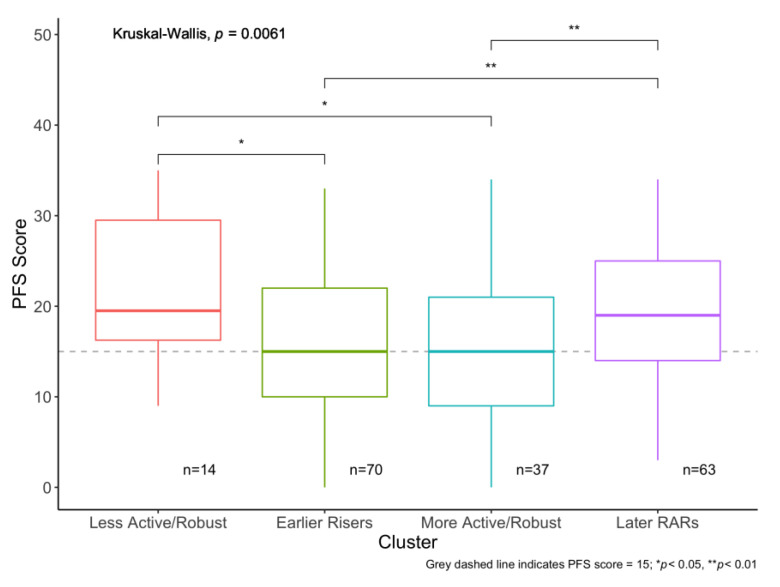
Rest–activity rhythm (RAR) profiles defined by k-means cluster analysis and their association with the PFS score. Differences in the Pittsburgh Fatigability Scale (PFS) Physical scores by cluster assignment. Overall differences tested with the Kruskal–Wallis test, and pairwise differences tested with two-sample Wilcoxon Rank Sum.

Figure 4 shows how distributions of PFS Physical scores differ by cluster assignment. Overall Kruskal–Wallis test indicates that the distributions of PFS Physical scores significantly differed across these clusters (*p* = 0.006). Pairwise two-sample Wilcoxon Rank Sum tests revealed that median PFS Physical scores in the “Less Active/Robust” were statistically significantly higher than those in “Earlier Risers” and “More Active/Robust” (*p* = 0.04, *p* = 0.04, respectively), and that “Later RARs” had higher median PFS Physical scores than “Earlier Risers” and “More Active/Robust” (*p* = 0.009 for both). “Earlier Risers” and “More Active/Robust” (*p* = 0.65) and “Less Active/Robust” and “Later RARs” (*p* = 0.45) did not differ significantly in PFS Physical scores.

Quantile regression results show that being in “Less Active/Robust” and “Late RARs” were associated with 6.14 (*p* = 0.05) and 3.53 (*p* = 0.03) point higher PFS Physical scores compared to “Earlier Riser” (the referent group), respectively (Table 4, Model 1). We chose the “Earlier Riser” as the referent group for two reasons. First, this group captures the largest proportion of the sample (*n* = 70) and therefore reflects the majority of participant’s RAR patterns. Secondly, results from individual models of RAR parameters suggested that earlier RAR timing was a protective feature, and thus other RAR profiles would represent similar or less-protective profiles. A likelihood ratio test (LRT) comparing the model with cluster assignments versus without shows that cluster assignments significantly improve model fit (*p* = 0.03). Additionally, having either “Less Active/Robust” or “Late RARs” was associated with 3.71 point (*p* = 0.01) higher PFS physical score compared to having either an “Earlier Riser” or “More Active/Robust” (Table 4, Model 2).

## 4. Discussion

Results of this cross-sectional study of objectively measured free-living physical activity in older adults suggest that later and more variable RARs were associated with greater perceived physical fatigability, as evidenced in the cluster analysis. This study also found that later activity patterns (i.e., those with later acrophases and up mesors) were associated with higher PFS physical scores, with each hour later being associated with a 1.29 or 1.38 point increase in PFS physical scores, respectively, and 46% increase in odds of having greater physical fatigability (PFS ≥ 15). These findings highlight the importance of RAR timing and are consistent with other studies, which have shown that later peaks in activity (e.g., specifically later acrophase) are associated with increased risk of a variety of negative health outcomes [16,17,18] and negative cognitive outcomes [31] in samples of older adults.

We also identified that higher beta values (e.g., more square-like RARs, or steeper transitions from rest to activity), were associated with higher physical PFS scores, however, this effect was quite small and difficult to interpret as the beta parameter was unitless. Prior studies have found mixed relationships between beta values and health in older adult samples. The Osteoporotic Fractures in Men Study (MrOS) found that lower beta values (less steep) were associated with higher risk of incident stroke [32]. Another study of older adult caregivers found that lower beta values were also associated with higher depressive symptom severity [33]. Both findings are in the opposite direction of what we saw in our work. The differences for these findings could be attributed to differences in cohorts (e.g., all male samples versus a largely female sample in the present study), or mechanistic differences. Smagula et al. [33] found that the effect of beta on depressive symptom severity of caregivers was strongly attenuated by the specific behaviors exhibited by those they cared for, suggesting that the demands of caregiving may have been the predominate driver determining the beta of the RAR. It is also plausible that our results may be driven by the “Less Active/Robust” group, as they had the largest betas, supporting the notion that these parameters are best interpreted jointly, rather than separately. It remains unclear exactly what role the beta parameter may play in determining risk for disease states and perceived physical fatigability.

The present study also found that those with greater physical fatigability (PFS ≥ 15) had overall dampened levels of physical activity across all time points, with higher physical activity between 4 a.m. and 8 a.m. significantly associated with lower physical PFS scores. These results are consistent with those found in Wanigatunga et al. [8], who found that BLSA participants with higher fatigability (based on the RPE scale) had an overall dampened activity pattern compared to those with lower fatigability, and that most pronounced differences were in the 8 a.m.–12 p.m. interval. While they did not see significant differences in the 4–8 a.m. time window, this could be attributed to differences between sample characteristics (the present cohort is older, more overweight and has reduced physical function), differences in the ascertainment of physical fatigability (RPE versus PFS) or differences in rise times in these samples. For example, we could speculate that differences in rise times (up mesor) may be the primary driver of the differences in mean levels of activity seen in the present study, as more highly fatigued participants may be delaying their rise time, resulting in dampened activity in their 4-8 am window. After adjusting for estimated rise time by using a rise-time adjusted “person time”, we still see that those with greater fatigability had lower activity levels, however, these results were not statistically significant (data not shown). This further corroborates the notion that timing of activity within the 24-h clock plays an important role in the relationship between physical activity and fatigability.

These results did not suggest an independent effect of variability of activity on perceived physical fatigability. We did not see a significant association of the pseudo-F of the total global variability of the RAR with perceived physical fatigability when tested independently. We also did not see a significant association of standard deviation of activity within 4-h time intervals with perceived physical fatigability. These results were not consistent with what we expected based on previous studies that found lower pseudo-F statistic (or less robust/rhythmic RARs) were associated with negative health outcomes in older adults [18,31]. However, none of these studies specifically explored the relationship between variability of activity and perceived physical fatigability, making the present study novel in this regard.

Most research surrounding the impact of sleep and RARs on health has explored each characteristic or feature separately. However, more recently, researchers are interested in understanding sleep and RARs as a multidimensional construct, in which features co-occur or work together to create a healthy (or unhealthy) RAR [34]. Using the k-means clustering algorithm, we aimed to capture the joint distribution of RAR features on parameters estimated from the extended cosine model. We identified two potentially high-risk profiles of RARs that were associated with greater perceived physical fatigability: “Later RARs” and “Less Active/Less Robust” RARs were both associated with higher PFS Physical scores. To date, no known studies have explored similar types of RAR “profiles” and related them to perceived physical fatigability. Nonetheless, these results are in line with what we might expect based on another study conducted by Smagula et al. [35], which used a similar clustering technique on RAR parameters and found that later and irregular RARs were associated with depression symptoms in a sample of adults [36].

As noted previously, we did not see an individual effect of variability (pseudo-F statistic) of the RAR associated with perceived physical fatigability; however, we saw that participants in high-risk RAR clusters with less robust/more variable RARs were more likely to have higher PFS Physical scores. This finding suggests that the independent effect of variability alone may not be as important as its joint effect alongside other features of the RAR. Interestingly, individuals in the “Less Active/Less Robust” cluster also had steeper betas, earlier acrophases and earlier down mesors. Based on models that tested these parameters separately, we might have expected that these RAR patterns with earlier acrophases would be associated with lower perceived physical fatigability. However, instead, we see that the joint effect of earlier timing, reduced activity, and variability may be indicative of a high-risk RAR profile. Of note, individuals with the “Less Active/Less Robust” pattern had earlier acrophases, but not earlier rise times (up mesor), suggesting that earlier rise times may play a distinctly important role as a protective factor against greater perceived physical fatigability. While we cannot know for certain without more information on sleep and activities, we might characterize those in the “Less Active/Less Robust” cluster as individuals who are highly fatigued. These individuals may compensate for their fatigue with delayed rise times, resulting in a steeper transition from rest to wake, shortened activity periods, and retiring to rest earlier in the day (earlier down mesors).

Limitations of the present study include its cross-sectional nature, which precludes our ability to understand if changes in RARs may influence changes in perceived physical fatigability. However, previous studies have shown that physical activity interventions may stabilize RARs in older adults [37], making physical activity a potential target for intervention to modify RARs. Future longitudinal studies should explore how changes in RARs are associated with changes in perceived physical fatigability. While the present study was able to capture the overall variability of the RAR, the pseudo-F statistic was not able to capture the timing of variability throughout the week. For example, low pseudo-F (or low stability) could be attributed to a participant waking up regularly throughout the night, or a participant who has systematic differences in their waking times (e.g., early riser during weekdays versus late riser on the weekend). Future studies could investigate the use of the residual circadian spectrum to quantify frequency domains of variability [36]. Additionally, there are limitations to the k-means clustering technique: it contains an implicit assumption of normality, or at least symmetry, of the data (through the use of means over other statistics of central tendency), and it is an unsupervised technique. Yet, sensitivity analyses showed that other clustering identification algorithms (such as those used in R’s statistical package mclust [38]) yielded the same number of clusters as the k-means. Future studies could explore the use of tree-based methods to classify RARs based on their association with perceived physical fatigability. Additionally, while sleep is deeply tied to RARs, these analyses do not explore the effect of sleep on the associations found here. However, this is beyond the scope of this paper as we were primarily focused on understanding the magnitude of daytime activity, timing of “getting going” and “slowing down”, and the variability of the entire circadian pattern, and not sleep per-se. A recent study found that shorter sleep time and more fragmented sleep was associated with greater perceived fatigability [39]. Finally, the present sample is also small and homogenous (largely white and women) and future studies should explore these relationships in larger, more diverse samples in order to determine the generalizability of these findings. 

One major strength of this study is that it is novel in its application of classic circadian rhythm research (RAR parameters) techniques to understanding perceived physical fatigability. It is also the first to utilize a clustering technique to identify RAR profiles associated with greater physical fatigability. Another strength of this study includes the use of objective accelerometry data, which limits the likelihood of biases present in self-reported physical activity measures. In addition, the use of a relatively healthy community-based sample suggests that our results may have broader clinical and public health implications. 

In conclusion, the findings of this study suggest that delayed, dampened and less robust RARs were associated with greater perceived physical fatigability in a community-based sample of older adults. This is the first study to investigate the role of RAR features on physical fatigability in older adults and highlights the importance of the circadian rhythm as a key factor in the relationship between physical activity and physical fatigability in older adults. This study provides evidence for future researchers and clinicians to focus intervention targets on specific profiles of magnitude, timing and variability of the RAR in order to stem the downward spiral into disability.

## Figures and Tables

**Table 1:** Characteristics of overall sample and stratified by Pittsburgh Fatigability Scale (PFS) perceived physical fatigability status.

Characteristics	Overall (*n* = 181) ^+^	PFS Physical Scores ≥15 (*n* = 111) ^+^	PFS Physical Scores <15 (*n* = 70) ^+^	*p* Value
MOVEUP Study	127 (70.2)	88 (79.3)	39 (55.7)	<0.001 *^,1^
Age, years	71.3 ± 6.7	70.9 ± 6.5	72.1 ± 6.9	0.20 *^,1^
Sex, female	143 (79.0)	92 (82.9)	51 (72.9)	0.11 *^,3^
Race, White	127 (70.2)	82 (73.9)	45 (64.3)	0.17 *^,3^
Education, ≥High School	140 (77.3)	88 (79.3)	52 (74.3)	0.43 *^,3^
Short Physical Performance Battery, 0–12	10.5 ± 1.8	10.3 ± 2.0	10.9 ± 1.5	0.02 *^,1^
Body mass index, kg/m^2^	32.3 ± 6.0	33.6 ± 5.6	30.3 ± 6.1	<0.001 *^,2^
Usual Gait Speed, m/s	1.0 ± 0.2	1.0 ± 0.2	1.1 ± 0.2	<0.001 *^,1^
Physical activity (CHAMPS) ^4^, MET-min/day	320.8 ± 251.7	292.7 ± 245.3	365.3 ± 257.0	0.03 *^,1^
Depression symptomology (CES-D) ^5^, 0–30	6.9 ± 5.9	8.0 ± 6.1	5.0 ± 5.2	<0.001 *^,1^

^+^ Mean ± SD, *n* (%); * *p* < 0.05. ^1^ Kruskal–Wallis rank sum test. ^2^ Two-sample *t*-test. ^3^ Pearson’s Chi-squared test. ^4^ CHAMPS: Community Healthy Activities Model Program for Seniors questionnaire. ^5^ CES-D: Center of Epidemiologic Studies Depression Scale.

**Table 2:** Individual associations of rest–activity rhythm (RAR) characteristics by the Pittsburgh Fatigability Scale (PFS) perceived physical fatigability status.

	Unadjusted Associations				Multivariable Adjusted Quantile Regression ^3^	
Characteristics	Overall (*n* = 181)^+^	PFS Physical Scores ≥ 15 (*n* = 111)^+^	PFS Physical Scores < 15 (*n* = 70)^+^	*p* Value	β Estimate (95% CI)	Standardized β (95% CI)
Alpha	−0.3 ± 0.3	−0.3 ± 0.3	−0.4 ± 0.2	0.380 ^1^	2.70 (−3.83, 9.23)	0.67 (−0.96, 2.30)
Beta	22.2 ± 50.1	26.0 ± 58.1	16.4 ± 33.5	0.120 ^1^	0.03 (0.01, 0.05) *	1.48 (0.10, 2.86) *
Acrophase	14.8 ± 1.3	14.9 ± 1.4	14.6 ± 1.1	0.020 *^,1^	1.29 (0.31, 2.27) *	1.67 (0.41, 2.92) *
Amplitude	5.9 ± 2.8	5.6 ± 2.7	6.4 ± 3.0	0.030 *^,1^	−0.09 (−0.58, 0.40)	−0.24 (−1.63, 1.15)
Mesor	2.8 ± 0.6	2.8 ± 0.6	2.9 ± 0.6	0.110 ^1^	−0.50 (−3.05, 2.05)	−0.30 (−1.86, 1.25)
Up Mesor (hours)	7.5 ± 1.3	7.7 ± 1.3	7.2 ± 1.2	0.004 *^,2^	1.38 (0.40, 2.36) *	1.76 (0.50, 3.01) *
Down Mesor (hours)	22.0 ± 2.0	22.1 ± 2.3	22.0 ± 1.6	0.250 ^1^	0.82 (−0.02, 1.66)	1.66 (−0.06, 3.37)
Pseudo-F Statistic	995.5 ± 479.6	962.8 ± 499.1	1047.4 ± 445.3	0.070 ^1^	0.00 (0.00, 0.00)	−0.17 (−1.56, 1.23)

^+^ Mean ± SD; * *p* < 0.05. ^1^ Kruskal–Wallis rank sum test. ^2^ Two-sample *t*-test. ^3^ Multivariable quantile regression models estimating median PFS score are adjusted for age, sex, race, body mass index, and depression symptomatology (CES-D score) (*n* = 177).

**Table 3:** Multivariate quantile regression models of mean daily activity and standard deviation of daily activity across four-hour time intervals *.

	Mean of Activity			Standard Deviation of Activity		
Clock Time	β Estimate	95% Confidence Interval	*p* Value	β Estimate	95% Confidence Interval	*p* Value
00:00–04:00	2.42	(−6.72, 11.56)	0.60	1.12	(−12.52, 14.76)	0.87
04:00–08:00	−4.50	(−8.39, −0.61)	0.03	−5.75	(−15.83, 4.33)	0.27
08:00–12:00	−3.05	(−6.98, 0.89)	0.13	−0.22	(−8.07, 7.64)	0.96
12:00–16:00	−1.96	(−5.83, 1.91)	0.32	−1.01	(−8.98, 6.95)	0.80
16:00–20:00	−1.53	(−5.82, 2.77)	0.49	−1.02	(−9.39, 7.35)	0.81
20:00–24:00	2.39	(−3.07, 7.84)	0.39	−7.42	(−16.64, 1.8)	0.12

* All models adjusted for age, sex, race, body mass index and depression symptomatology score.

**Table 4:** Multivariable quantile regression of effects of rest–activity rhythm (RARs) profiles on median Pittsburgh Fatigability Scale Physical scores *.

	β Coefficient	95% Confidence Interval	*p*-Value	LRT ^+^
Model 1				
Earlier Risers	ref			0.03
More Active/Robust	0.45	(−2.78, 3.68)	0.79	
Later RAR	3.53	(0.30, 6.76)	0.03	
Less Active/Robust	6.14	(−0.01, 12.29)	0.05	
Model 2				
Earlier Risers or More Active/Robust	ref			
Less Active/Robust or Later RAR	3.71	(0.99, 6.43)	0.01	

* All models adjusted for age, sex, race, body mass index and depression symptomatology score. ^+^ LRT = likelihood ratio test for comparing model with cluster assignments versus model without.

## Data Availability

The data presented in this study are available on request from the corresponding author.

## Supplementary Materials

The following are available online at https://www.mdpi.com/1424-8220/21/5/1718/s1, Table S1. Cohen’s D effect sizes for demographic and RAR characteristics based on Pittsburgh fatigability scale (PFS) perceived physical fatigability status (PFS physical scores ≥ 15 versus PFS physical scores < 15); Figure S1. k-means clustering diagnostics for 4 clusters.
