# Supplementary material for: Profiles of Accelerometry-Derived Physical Activity Are Related to Perceived Physical Fatigability in Older Adults
Source: Sensors (Basel). 2021 Mar 2;21(5):1718. doi: 10.3390/s21051718 (PMC7958607; doi:10.3390/s21051718)
Supplement: Supplementary file 1 [file sensors-21-01718-s001.pdf]

Supplemental Material

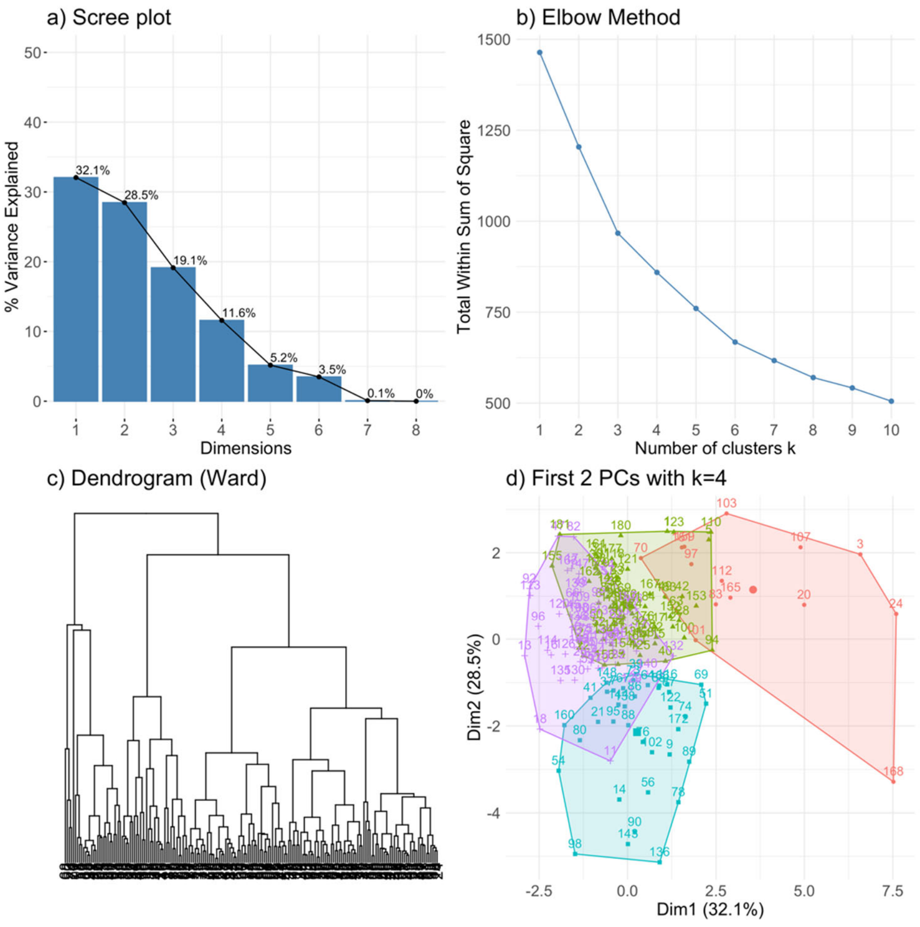

Figure S1. k-means clustering diagnostics for 4 clusters.

a) percent of variance explained from principal components analysis (PCA); b) “Elbow method” of within sum of squares estimated by different cluster k assignments; c) Ward method dendrogram; d): visualization of first two dimension of PCA analysis with cluster assignments overlaid.

Table S1. Cohen's D effect sizes for demographic and RAR characteristics based on Pittsburgh Fatigability Scale (PFS) perceived physical fatigability status (PFS Physical Score ≥15 versus PFS Physical Score <15).

	**Cohen's D Effect Size (95% CI)**
**Demographic Characteristics**	
Age	−0.18 (−0.48, 0.12)
Short Physical Performance Battery	−0.31 (−0.62, 0.00)
Body mass index, kg/m^2	0.57 (0.26, 0.88)
Usual Gait Speed, m/s	−0.88 (−1.21, −0.54)
Physical activity (CHAMPS), MET-min/day	−0.29 (−0.60, 0.01)
Depression symptomology (CES-D), 0-30	0.52 (0.20, 0.83)
**RAR characteristics**	
# of observation days	−0.50 (−0.81, −0.19)
Alpha	0.23 (−0.08, 0.53)
Beta	0.19 (−0.11, 0.49)
Acrophase	0.25 (−0.06, 0.55)
Amplitude	−0.27 (−0.57, 0.03)
Mesor	−0.19 (−0.49, 0.11)
Up Mesor (hours)	0.45 (0.14, 0.76)
Down Mesor (hours)	0.04 (−0.26, 0.33)
Pseudo-F Statistic	−0.18 (−0.48, 0.12)
